# Effects of the intradiscal implantation of stromal vascular fraction plus platelet rich plasma in patients with degenerative disc disease

**DOI:** 10.1186/s12967-016-1109-0

**Published:** 2017-01-13

**Authors:** Comella Kristin, Silbert Robert, Parlo Michelle

**Affiliations:** 1 US Stem Cell Inc, 13794 NW 4th Street, Suite 212, Sunrise, FL 33325 USA; 2 PM&R Associates, 6640 Parkdale Place, Indianapolis, IN 46254 USA

**Keywords:** Stromal vascular fraction (SVF), Platelet rich plasma (PRP), Adipose derived stromal/stem cells (ADSCs), Stem cells, Adipose tissue, Degenerative disc disease, Back pain, Cell therapy

## Abstract

**Background:**

Stromal vascular fraction (SVF) can easily be obtained from a mini-lipoaspirate procedure of fat tissue and platelet rich plasma (PRP) can be obtained from peripheral blood. The SVF contains a mixture of cells including ADSCs and growth factors and has been depleted of the adipocyte (fat cell) population. We evaluated the safety and efficacy of administering SVF and PRP intra-discally into patients with degenerative disc disease.

**Methods:**

A total of 15 patients underwent a local tumescent liposuction procedure to remove approximately 60 ml of fat tissue. The fat was separated to isolate the SVF and the cells were delivered into the disc nucleus of patients with degenerative disc disease. The subjects were then monitored for adverse events, range of motion, visual analog scale (VAS), present pain intensity (PPI), Oswestry Disability Index (ODI), Beck Depression Inventory (BDI), Dallas Pain Questionnaire and Short Form (SF)-12 scores over a period of 6 months. Safety events were followed for 12 months.

**Results:**

No severe adverse events (SAEs) were reported during a 12 month follow up period with no incidences of infection. Patients demonstrated statistically significant improvements in several parameters including flexion, pain ratings, VAS, PPI, and short form questionnaires. In addition, both ODI and BDI data was trending positive and a majority of patients reported improvements in their Dallas Pain Questionnaire scores.

**Conclusions:**

Overall, patients were pleased with the treatment results. More importantly, the procedure demonstrated a strong safety profile with no severe adverse events or complications linked to the therapy.

*Trial registration* NCT02097862. Name of registry: www.clinicaltrials.gov. https://clinicaltrials.gov/ct2/show/NCT02097862?term=bioheart&rank=6. Date of registration: March 25, 2014; Date of enrollment: March 2014

## Background

Degenerative disc disease (DDD) is a condition associated with the degeneration of one or more of the discs in the spine. DDD can cause severe chronic pain in the low back which can radiate to the hips and legs. There can be severe inflammation and degeneration of the fibrocartilage.

Recent studies have focused on the use of adult stem cells for disorders such as degenerative disc disease. Mesenchymal stem cells (MSCs) are non-hematopoietic, multipotent progenitor cells, which can be isolated from various human adult tissues. The potential to form cells of multi-lineages has indicated the potential of these cells in cases of degenerative disc disease. In recent years, MSCs have been shown to possess broad range of regenerative capabilities, modulating disease progression repairing lesions so closely associated with degenerative disc disease [1, 2]. MSCs are being investigated as a regenerative biologic agent because of their ability to differentiate into multiple tissue types and to self-renew.

The paracrine activity of MSCs is thought to be one of the major means by which these cells mediate anti-inflammatory, anti-apoptotic, anti-fibrotic, angiogenic, mitogenic and wound healing properties. The complex interplay of the biological mediators secreted by MSCs has been shown to be important in regulating regeneration of damaged or diseased organs and tissues of the body. It has also been shown that the pre-curser to the MSC is the pericyte which are the cells present on the microvessels and capillaries throughout the body. These cells become “activated” when an injury is recognized and detach to become medicinal MSCs. An immune-modulatory effect is initiated where other cells are called to help with the healing process while other secreted molecules will establish a regenerative microenvironment by setting up a trophic field [3].

Recent studies have identified adipose tissue as a new source of mesenchymal stem cells. As lipoaspiration provides relatively simple access to this stem cell pool, and with the large numbers of cells present in adipose tissue, its future potential as a stem cell reservoir for degenerative disc disease is promising.

Stem cells derived from a patient’s own fat are referred to as adipose-derived stem cells [4]. Adipose-derived stem cells or ADSCs are multi-potential in that they have the ability to differentiate into a variety of different types of tissue including but not limited to bone, cartilage, muscle, and fat. These cells have also been shown to express a variety of different growth factors and signaling molecules (cytokines), which recruit other stem cells to facilitate repair and healing of the affected tissue. ADSCs are very angiogenic in nature and can promote the growth of new blood vessels. In addition, ADSCs might play a role in the local inflammatory process [5, 6].

A stromal vascular fraction (SVF) can easily be isolated from fat tissue in approximately 30–90 min in a clinic setting using a mini-lipoaspirate technique. The SVF contains a mixture of cells including ADSCs and growth factors and has been depleted of the adipocyte (fat cell) population. It has been shown that cells isolated from the SVF contain an abundance of CD34+ cells [7]. This marker is present on both pericytes and mesenchymal cells. Cells expressing CD34 are also known to reside in a periendothelial location and stabilize endothelial networks. SVF can be used in a point of care setting for a variety of indications and is currently being used in thousands of clinics world-wide with varying degrees of success reported. Adipose tissue is quickly becoming the preferred source for point of care treatments in clinic due to the high number of MSCs that can be obtained and the low number of leukocytes as compared to bone marrow [8]. In addition, adipose tissue has a significantly higher amount of pericytes which are the precursors to MSCs [9, 10].

Stem cells from adipose tissue offer a novel therapy for patients with degenerative disc disease. SVF injected directly into the disc may reduce inflammation and promote healing. SVF is an attractive therapeutic method given that the harvesting process is safe and the cells are readily available in usually large quantities. In Vitro studies have demonstrated that ADSCs are able to stimulate matrix synthesis and cell proliferation of degenerated nucleus pulpous cells. This may promote the restoration of damaged nucleus cells in a degenerated disc [11]. In a murine model of chronic disc degeneration, ADSC transplantation promoted new expression of proteoglycans and increased levels of aggrecan [12]. ADSCs were also studied in a rabbit model of degenerative disc disease. The ADSC injected discs exhibited elevated extracellular matrix and minimal ossification as compared to the control discs [13].

Platelet rich plasma is a mixture of growth factors and fibrin obtained from autologous peripheral blood. PRP has been utilized in a variety of musculoskeletal indications with evidence of anti-inflammatory and healing properties. One study demonstrated that intradiscal injections of PRP led to symptomatic clinical benefit in individuals with chronic discogenic lower back pain [14]. By combining PRP with SVF, there may be an increased number of growth factors anad proteins which could translate to improved patient outcomes.

Pettine, et al. have reported on the use of bone marrow concentrate to treat discogenic pain as an alternative to surgery. The cells were injected directly into the nucleus pulposus with no complications reported. There was a 71% reduction in VAS pain scales and ODI improvements through 2 years [15]. In another pilot study, patients with degenerative disc disease were injected into the nucleus pulpous area with culture expanded autologous bone marrow MSCs. Patients exhibited rapid improvement of pain and disability and elevated water content in the disc at the 12 month follow up [16]. We report the safety and preliminary efficacy results of intradiscal SVF and PRP administration in patients with degenerative disc disease.

## Methods

### Study design

The open label study was conducted on 15 patients with degenerative disc disease. The protocol was approved by the institutional review board and all patients provided written informed consent. SVF was injected directly into the nucleus pulpous under fluoroscopic guidance. Clinical evaluations were scheduled at baseline, 2, and 6 months. The primary safety endpoint was serious adverse events (SAEs) and were defined as any event that was fatal or life-threatening, led to hospitalizations, or required major medical intervention. Physical parameters including height, weight, blood pressure, pulse and low back range of motion as measured by inclinometer were collected. Patients were evaluated for visual analog score (VAS), present pain index (PPI), and minimum, maximum, and current pain ratings. Psychological data was also collected including Beck Depression Inventory (BDI), Oswestry Disability Index (ODI), Short Form McGill Pain Questionnaire (SM-MPQ), Short Form 12 (SF-12), and the Dallas Pain Questionnaire.

### Patient eligibility

Patients age 18–90 years with degenerative disease of one, two or three lumbar discs with predominant back pain after conservative treatment (physical and medical) for over 6 months were eligible for the study. Patients must have a fibrous ring capable of holding the cell implantation as demonstrated by MRI image. Patients with congenital or acquired diseases leading to spinal deformations, active cancer or infections including human immunodeficiency virus, hepatitis B or C, or cytomegalovirus were excluded. In addition, patients with spinal segmental instability, spinal canal stenosis, isthmus pathology, more than 50% loss of height, or modic III changes on MRI images were also excluded from the study.

### Cell preparation and study intervention

From each patient, approximately 60 ml fat was collected using a 3 mm aspiration cannula with prior administration of tumescent solution. The tissue was prepared using an adipose stromal vascular fraction preparation kit (US Stem Cell, Inc. Sunrise, FL). The adipose tissue was washed with buffered saline and digested using collagenase (Cellase, US Stem Cell, Inc., Sunrise, FL) at 37 °C for 12–15 min with agitation at 5-minute intervals. The suspension was centrifuged at 1200×*g* for 5 min to collect the SVF as a pellet. The pellet was washed twice and filtered through a 100 μm cell strainer with buffered saline to remove any residual enzyme. The final SVF pellet was resuspended in approximately 1–3 ccs of autologous platelet rich plasma (PRP). PRP was prepared by collecting autologous peripheral blood and centrifuging at 500 g for 8 min.

Patients placed in a prone position on the fluoroscopy table. Standard sterile preparation and local anesthesia was administered. Anteroposterior and lateral fluoroscopic imaging was utilized to confirm proper needle position in the nuclei of the disc. A small amount of contrast agent was injected followed by approximately 1 cc of SVF/PRP suspension. If more than one disc was symptomatic, the SVF was divided and prepared with approximately 1 cc of PRP.

### Outcomes

The primary safety outcome was the incidence of SAEs over 6 months. The efficacy outcomes included visual analog score (VAS), present pain index (PPI), and minimum, maximum, and current pain ratings. Psychological data was also collected including Beck Depression Inventory (BDI), Oswestry Disability Index (ODI), Short Form McGill Pain Questionnaire (SM-MPQ), Short Form 12 (SF-12), and the Dallas Pain Questionnaire. This study was designed to primarily assess the safety and feasibility of the intradiscal SVF transplantation procedure and secondarily to provide preliminary data regarding the efficacy of intradiscal SVF transplantation. Formal power calculations were not performed. Paired *T* test statistical analyses were performed and confidence intervals are presented with 95% degree of confidence. All statistical tests used a significance level of α ≤ 0.05.

## Results

### Patient baseline evaluation

A total of 15 patients were enrolled in the study and treated at one clinical site. Seventy-three percent of the patients were male and 27% female with an average age of 51.5 (range 32–76). A total of 15 patients completed follow up at 2 and 6 months.

### Adipose and SVF collection

**Fig. 1:**
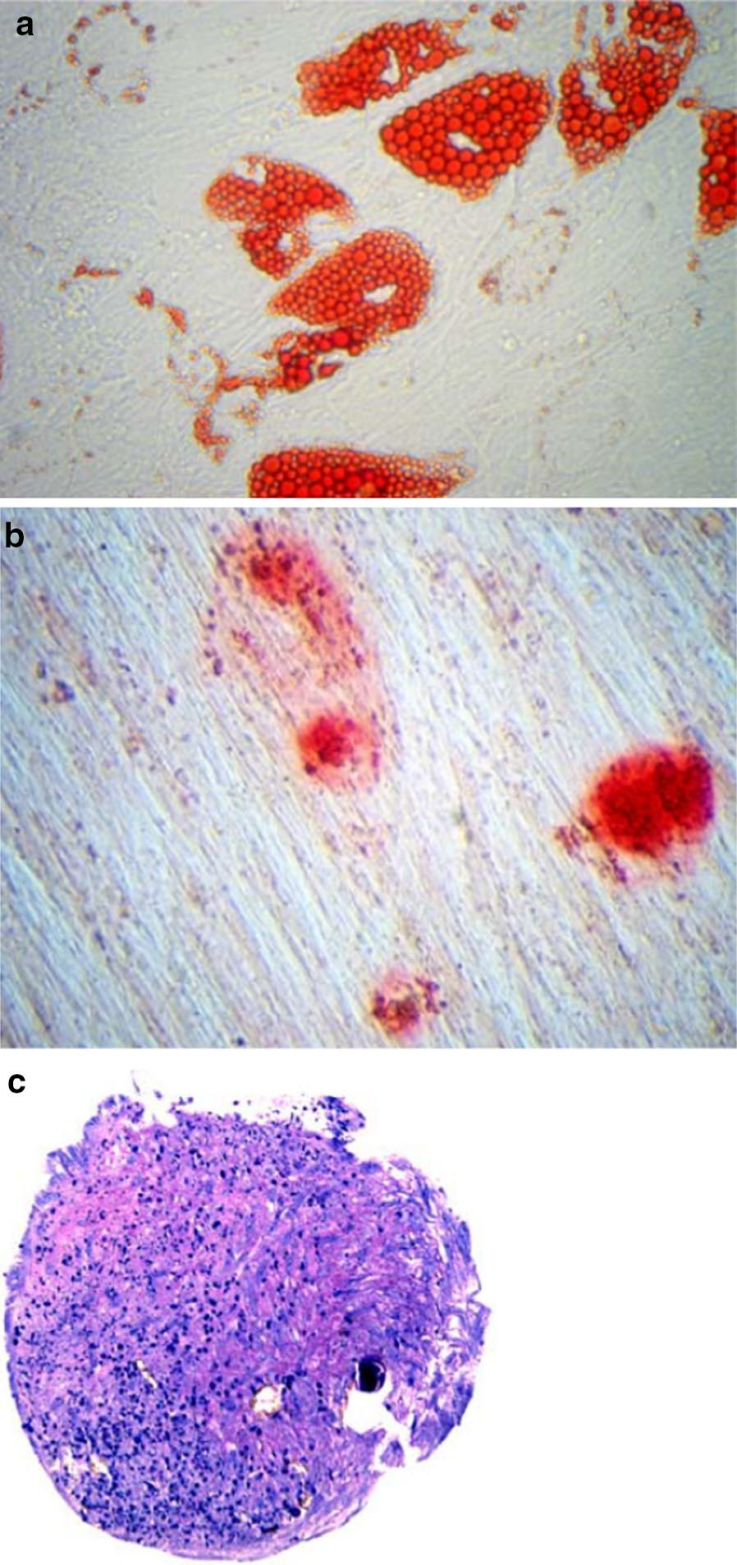
**a** Adipogenesis fat differentiation (Oil Red-O), **b** osteogenesis bone differentiation (Alizarin *Red* S), **c** chondrogenic cartilage differentiation (toluidine blue sodium borate stain)

All procedures were well tolerated and uneventful, resulting in a collection of approximately 60 ccs of adipose tissue. Approximately 30–60 million SVF cells were obtained. According to validation studies, the population obtained is at least 50% positive for CD34 with a viability of greater than 90% (data not shown). In addition to testing the proliferation capacity of the cells, differentiation assays for adipogenesis (fat), osteogenesis (Bone) and chondrogenesis (cartilage) were completed. The samples shown in Fig. 1 displayed positive differentiation results confirming the presence of multi-potential mesenchymal stem cells.

### Transplantation procedure

The transplantation procedure was successful in 15/15 patients. Patients received approximately 30–60 million cells in 1–3 ccs volume of PRP. A volume of approximately 1 cc of cells in PRP were placed directly into the nuclei of each disc as determined by fluoroscopy.

### Efficacy outcomes

**Fig. 2:**
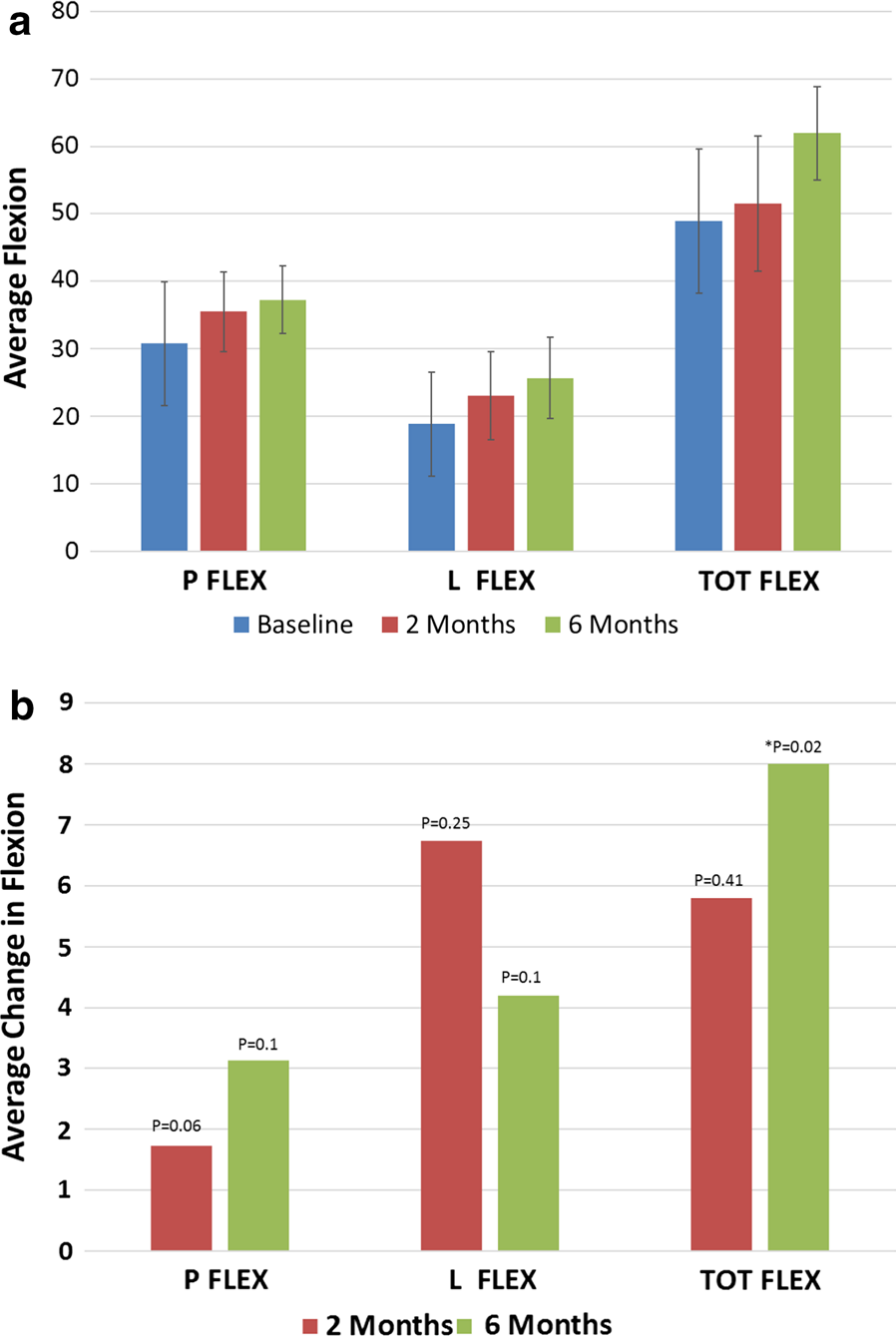
**a** Average flexion, **b** average change in flexion

Patients demonstrated improvements in flexion over the 6 month follow up period (Fig. 2). Figure 2a shows the average flexion at baseline, 2 and 6 months post injection. Patients demonstrated positive trends with an increase in pelvic, lumbar and total flexion over the 6 months period. Figure 2b shows the average change in flexion over baseline at both 2 and 6 months. All data points were trending in the positive direction with a statistically significant (p = 0.02) improvement in total flexion at 6 months.

**Fig. 3:**
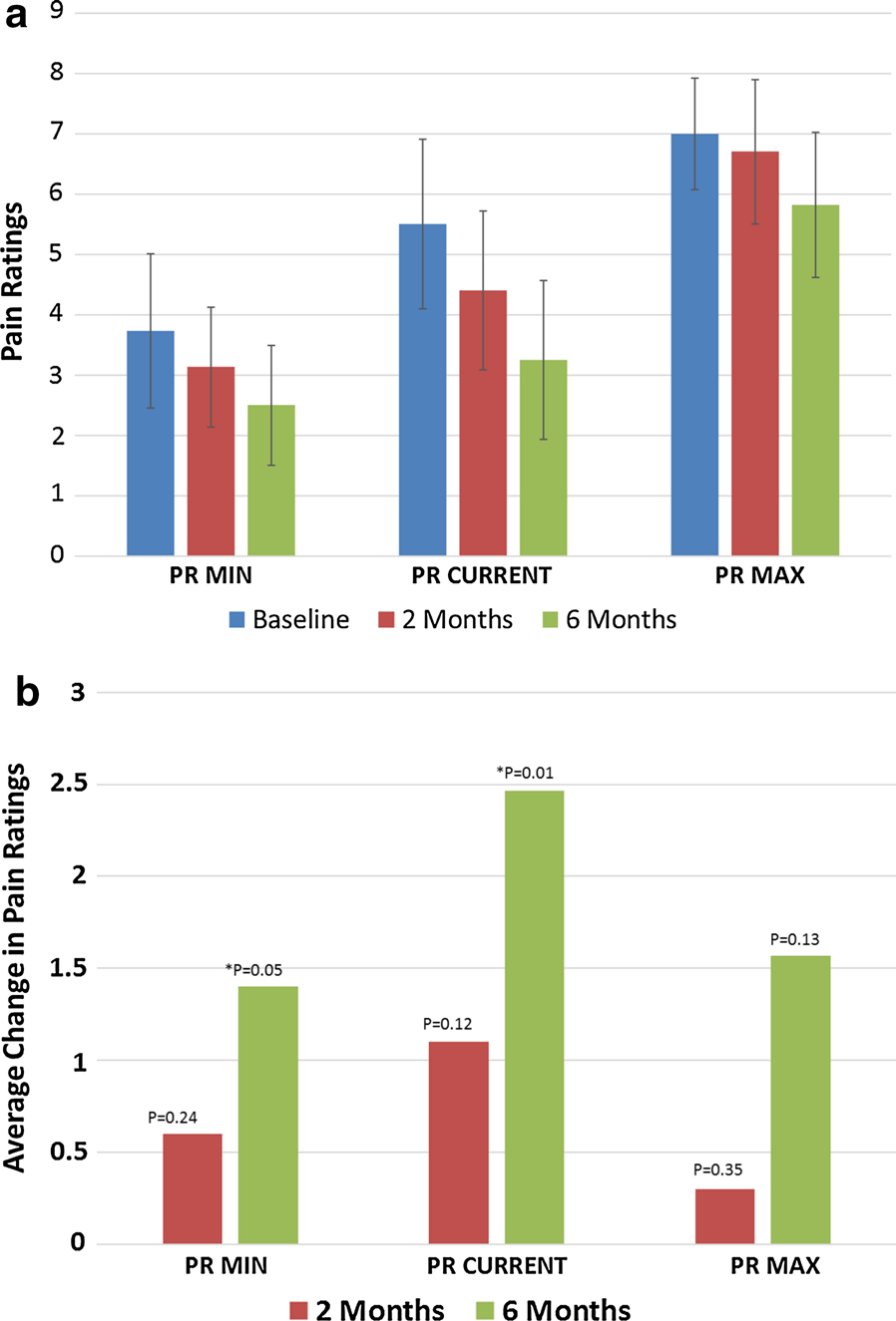
**a** Average pain ratings, **b** average change in pain ratings

Figure 3 shows the pain ratings over the 6 month follow up period. Figure 3a is the Average Pain ratings at baseline, 2 and 6 months post injection. Patients experienced a reduction in pain from baseline to 2 months and again from 2 to 6 months. Figure 3b displays the average change in pain ratings at 2 and 6 months over baseline. The majority of patients (10) were reporting less pain at 6 months post injection as compared to baseline. Both the minimum and current pain ratings scores showed statistically significant improvements (p = 0.05 and p = 0.01 respectively).

**Fig. 4:**
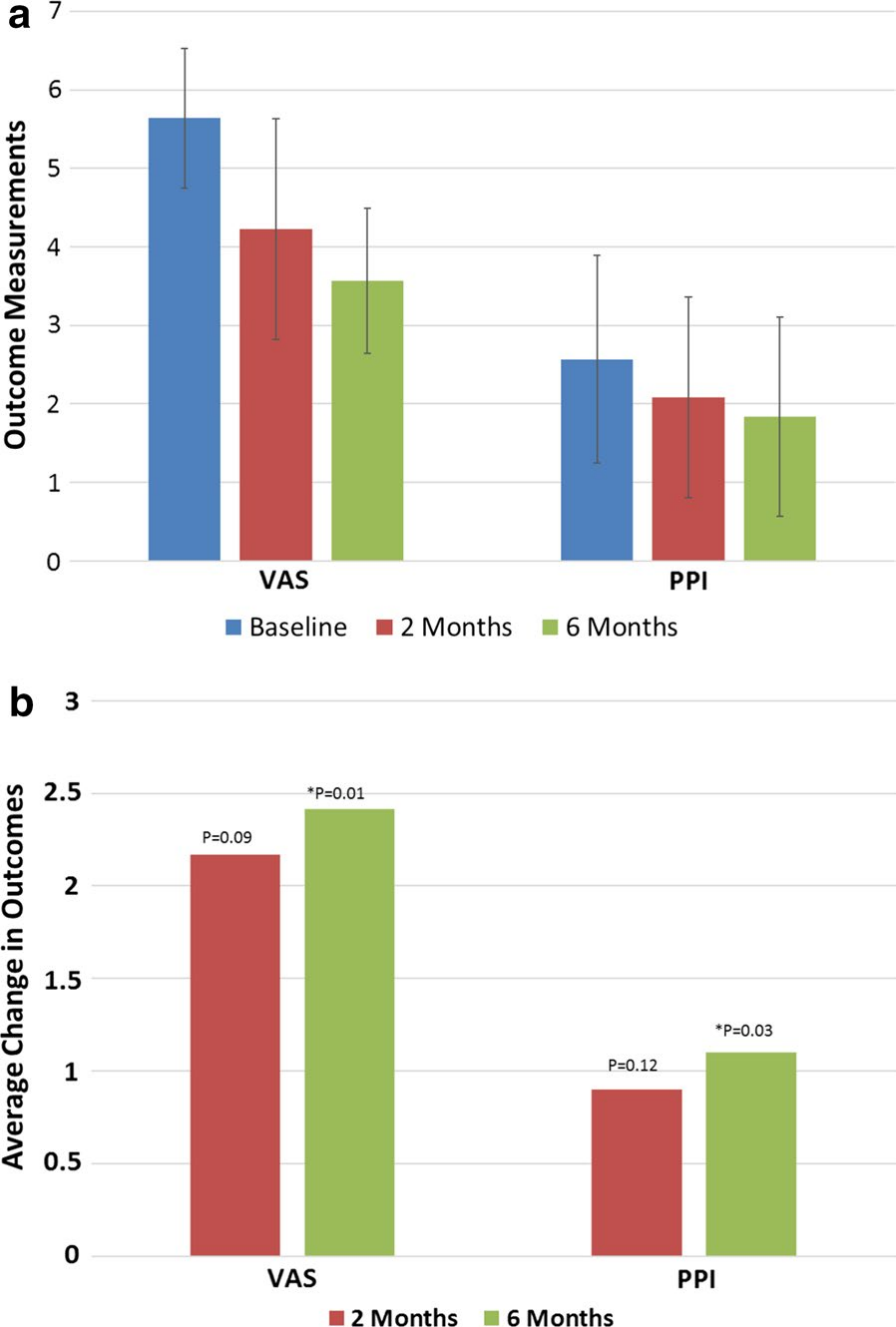
**a** Average visual analog score and present pain index, **b** Average change in VAS and PPI

Patients reported a reduction in pain and discomfort according to both VAS and PPI (Fig. 4). VAS scores statistically improved from an average of 5.6 at baseline to 3.6 at 6 months (p = 0.01). PPI scores statistically improved from an average of 2.6 at baseline to 1.8 at 6 months (p = 0.03). Figure 4b shows the average improvement in outcome scores for VAS and PPI.

**Fig. 5:**
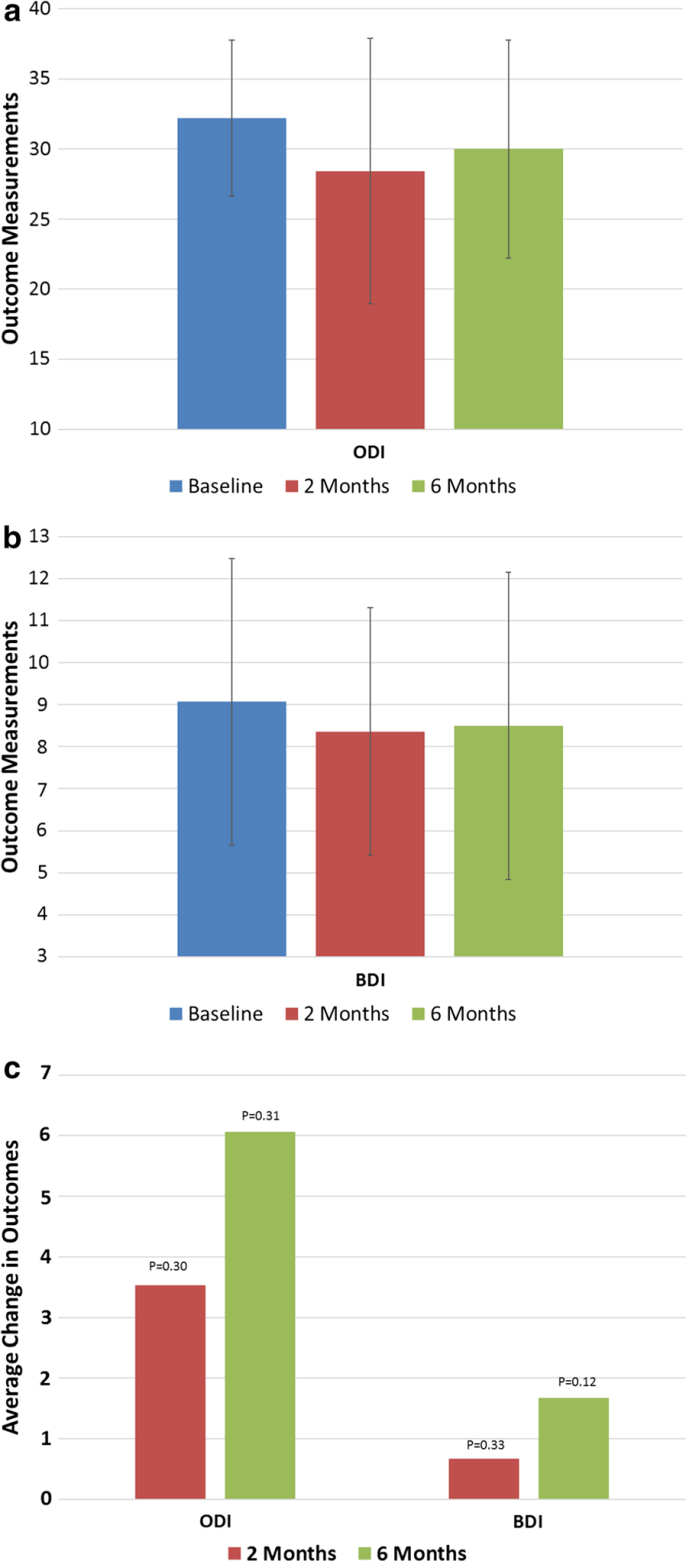
**a** Average oswestry disability index, **b** average beck depression inventory, **c** average change in ODI and BDI

Psychological data ODI and BDI results displayed a modest improvement (Fig. 5a, b). The data did not reach statistical significance at either 2 months or 6 months (Fig. 5c).

**Fig. 6:**
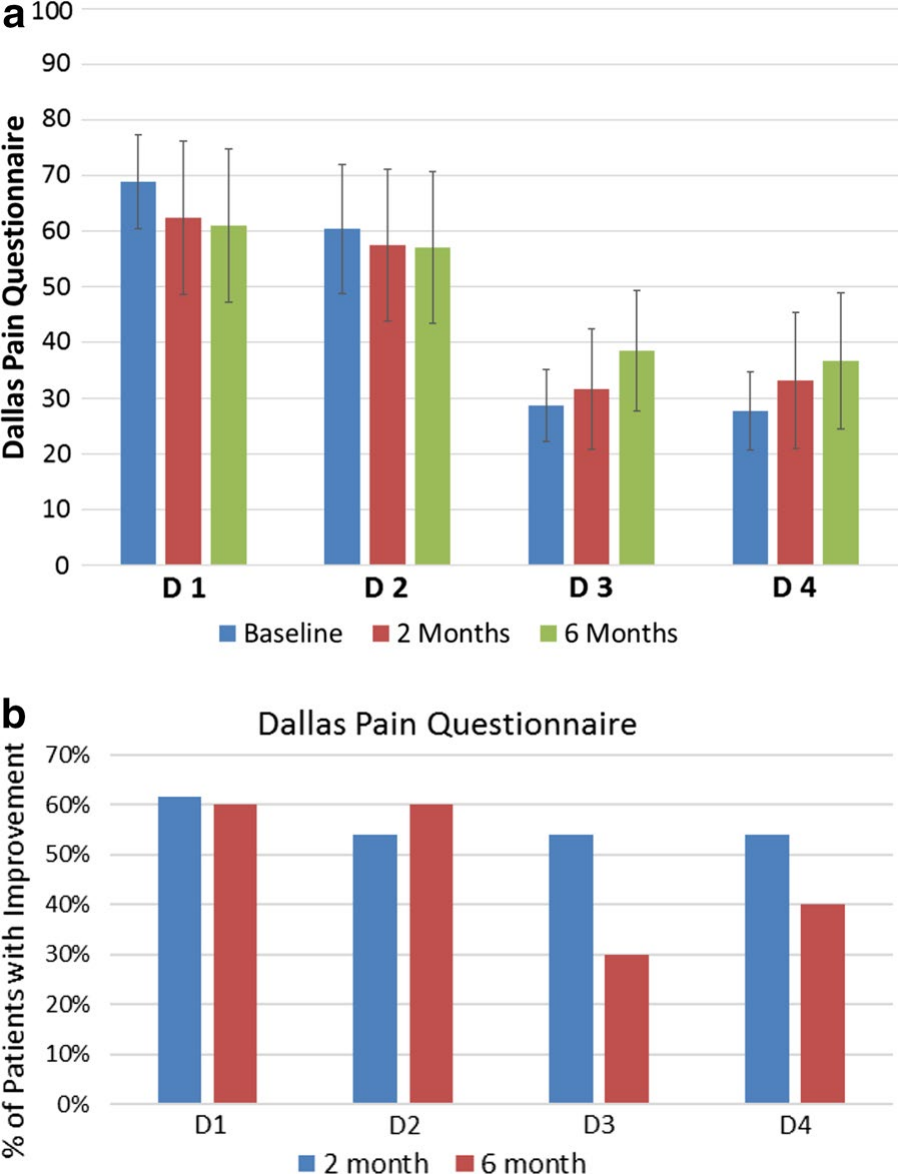
**a** Average dallas pain questionnaire results, **b** percentage of patients with improvements in dallas pain questionnaire

The Dallas Pain Questionnaire results are shown in Fig. 6. There are 16 questions which are divided into four sections including: D1 daily activities, D2 work/leisure activities, D3 anxiety/depression, and D4 social interest. Figure 6a shows the values of the four sections at baseline, 2 and 6 months. On average, patients demonstrated a slight improvement in their daily activities and work/leisure activities at both 2 and 6 months. Patients, however, demonstrated a slight increase in anxiety/depression and reduction in social interest. Figure 6b shows the percentage of patients that improved in each parameter of the questionnaire. A majority of patients showed improvements at both 2 and 6 months for daily activities and work/leisure activities. In addition, a majority of patients showed improvements in anxiety and social interest at 2 months and 30 and 40% respectively showed improvements at 6 months.

**Fig. 7:**
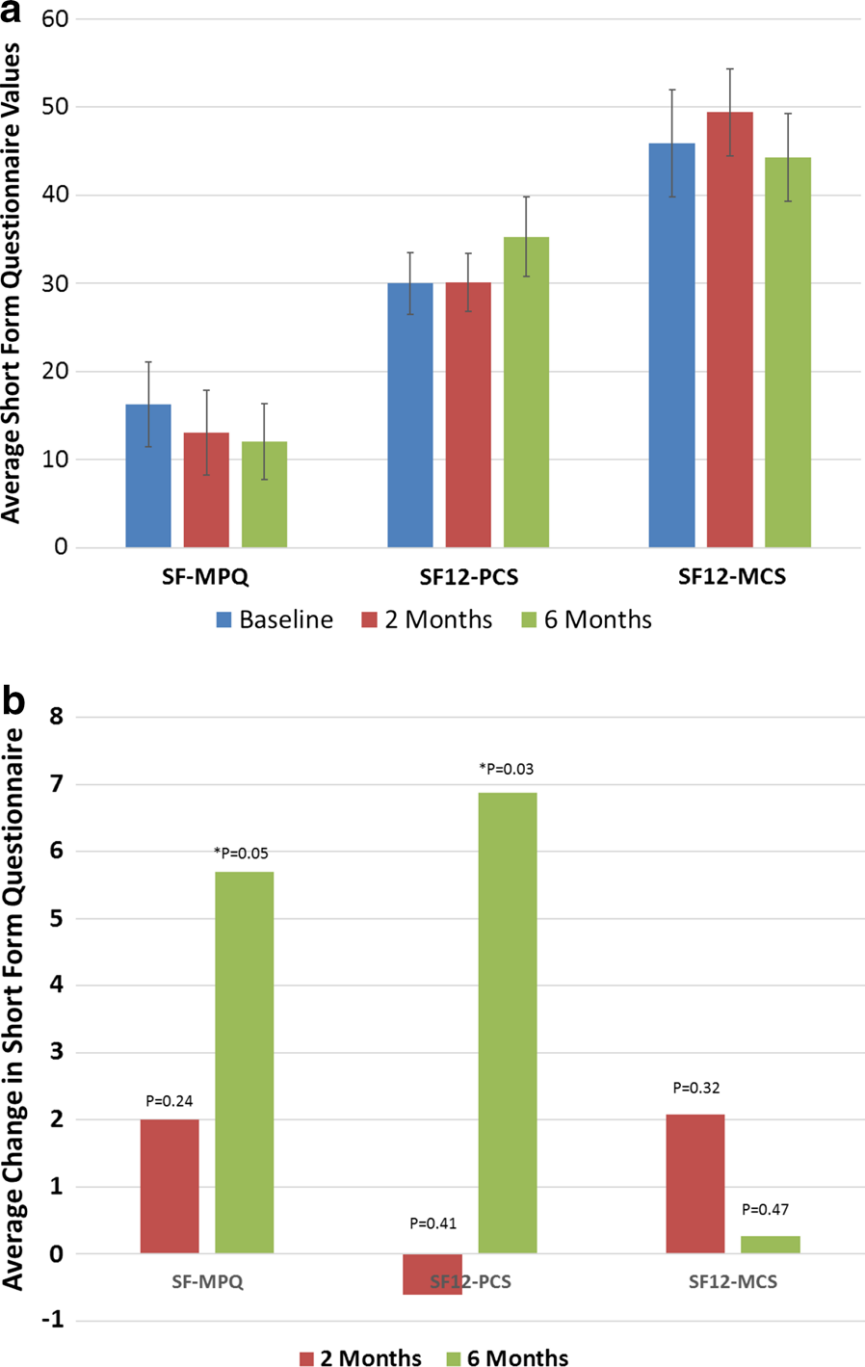
**a** Average short form questionnaire results, **b** average change in short form questionnaire

Results for both the Short Form McGill Pain Questionnaire and the Short Form 12 are shown in Fig. 7. The SF-MPQ provides valuable information on the sensory, affective and evaluative dimensions of pain experience with higher numbers indicating more pain and lower numbers indicating less pain. The SF12 is a generic measure that can provide glimpses into mental and physical functioning and overall health-related-quality of life to provide easily interpretable scales for physical and mental health. Physical and mental health composite scores (PCS & MCS) are computed using the scores of twelve questions, where a zero score indicates the lowest level of health and 100 indicates the highest level of health. Figure 7a shows the average short form questionnaire values and 7b shows the average change/improvement from baseline. Patients reported a slight improvement in SF-MPQ and a statistically significant improvement at 6 months (p = 0.05). Patients also reported a significant improvement in SF12-PCS at 6 months (p = 0.03). No significant changes in SF12 at either time point were observed.

### Safety analysis

No severe adverse events (SAEs) were reported during a 12 month follow up period. Other reported events included soreness in the abdomen after the mini-liposuction procedure or soreness in the back after injections. Patients were instructed to take previously prescribed opioids for pain and all events resolved within 7–10 days. All occurrences resolved without intervention. There were no incidences of infection.

## Discussion

Degenerative disc disease is associated with symptoms of pain and possibly radiating weakness or numbness with limited clinical options. Until recently, patients had few options. Surgery requires extensive recovery, and time away from work, usually at least 6 weeks. Also, the risk of complications is significant. Aside from the complications that might arise from anesthesia, there is a 1 in 10,000 risk of bowel or bladder incontinence and a 1 in 1000 risk of nerve root damage. There may also be 1–3% risk of CSF leak, 1% infection risk and 5–10% risk of spinal instability [17].

In recent years, stem cell therapy has developed with promising preclinical results and preliminary clinical results. Adipose derived stem cells as part of the stromal vascular fraction are a feasible candidate for degenerative indications. The SVF does not require in vitro culture expansion and is easy to collect bedside. These cells can be placed directly into the nuclei of the disc utilizing a minimally invasive injection technique guided by fluoroscopy.

This clinical study demonstrated the safety and feasibility of utilizing the SVF in degenerative disc patients. No major safety issues were noted and the procedures were well tolerated in all patients. In addition, patients demonstrated statistically significant improvements in several parameters including flexion, pain ratings, VAS, PPI, and short form questionnaires. Although ODI and BDI did not show statistically significant changes due to the low number of subjects in the trial, the data was trending positive. In addition, a majority of patients reported improvements in their Dallas Pain Questionnaire scores.

Although this study suggests that the use of SVF is safe and feasible, the general under powering of the study coupled with the lack of placebo control would render additional studies necessary to determine the true clinical effect of the treatment. In addition, several patients were lost to follow up which could compound the data and create patient bias. Given the encouraging results on this small sample size with statistical significance, large appropriately powered clinical studies blinded to both clinical staff and patients are warranted.

## Conclusion

The current study sought to define the safety and feasibility of intradiscal transplantation of autologous SVF in patients with degenerative disc disease. Several parameters demonstrated statistically significant improvements over a 6 month time period. A true evaluation of efficacy and safety would require larger phase II/III studies. However, the current study does provide encouraging feasibility data regarding the intradiscal stem cell treatment and suggests some clinical benefit of the SVF therapy in degenerative disc patients.

## Abbreviations

SVF: stromal vascular fraction
PRP: platelet rich plasma
ADSCs: adipose derived stem/stromal cells
MSC: mesenchymal stem cell
SAE: severe adverse event
VAS: visual analog score
PPI: present pain index
ODI: oswestry disability index
BDI: beck depression inventory
SF-MPQ: short form McGill pain questionnaire
SF12: short form 12
MCS: mental health composite score
PCS: physical health composite score
