# Erratum to: Effects of the intradiscal implantation of stromal vascular fraction plus platelet rich plasma in patients with degenerative disc disease

**DOI:** 10.1186/s12967-017-1217-5

**Published:** 2017-05-22

**Authors:** Kristin Comella, Robert Silbert, Michelle Parlo

**Affiliations:** 1 US Stem Cell Inc, 13794 NW 4th Street, Suite 212, Sunrise, FL 33325 USA; 2 PM&R Associates, 6640 Parkdale Place, Indianapolis, IN 46254 USA

## Erratum to: J Transl Med (2017) 15:12 DOI 10.1186/s12967-016-1109-0

In the original version of this article [1], published on 13 January 2017, the author names have been displayed in the wrong order. In this Erratum the incorrect names and correct names are shown. The original publication of this article has been corrected.

Originally the author names have been published as:

- Comella Kristin
- Silbert Robert
- Parlo Michelle

The correct names are:

- Kristin Comella
- Robert Silbert
- Michelle Parlo

## References

[1] Comella K, Silbert R, Parlo M (2017). Effects of the intradiscal implantation of stromal vascular fraction plus platelet rich plasma in patients with degenerative disc disease. J Transl Med.

